# Supplementary material for: A Likelihood Approach for Real-Time Calibration of Stochastic Compartmental Epidemic Models
Source: PLoS Comput Biol. 2017 Jan 17;13(1):e1005257. doi: 10.1371/journal.pcbi.1005257 (PMC5240920; doi:10.1371/journal.pcbi.1005257)
Supplement: S1 File — (TAR.GZ) [file pcbi.1005257.s014.tar.gz › HSPH_Online-SI-Revision/PF08/n1-severe/n1-severe_PF_table-4.pdf]

1	R0	{1.51081, 13.7078}	{1.65262, 14.5487}	{{1.1961, 8.24622}, {2.16131, 20.}}	{21.1833, 26.633}	inf	45.	77.1002	{26.8041, 141.528}	93.9198	0.259864	newly	25.	37.16	{14., 67.}	74.08	0.242404	Reff	1.25397	1.06086	{0.652149, 1.35155}	20.6845	ar	656.	746.11	{501., 978.}	25.6905
2	R0	{1.73182, 11.3873}	{1.75008, 13.9731}	{{1.23764, 6.66802}, {2.39258, 19.9338}}	{22.7037, 41.0931}	inf	37.	91.6842	{26.4923, 192.378}	160.615	0.356946	newly	33.	48.06	{17., 82.}	71.5758	0.238015	Reff	1.5621	1.23964	{0.836693, 1.67614}	26.9032	ar	653.	770.51	{475., 1018.}	30.0107
3	R0	{1.50449, 10.3474}	{1.74269, 14.174}	{{1.23585, 8.13391}, {2.4749, 20.}}	{25.3926, 47.328}	inf	32.	85.4285	{25.8838, 173.019}	174.085	0.354033	newly	23.	43.	{14., 76.}	104.522	0.292857	Reff	1.30891	1.12499	{0.689362, 1.46091}	23.4181	ar	651.	772.85	{526., 1016.}	29.2089
4	R0	{1.63493, 9.1955}	{1.84211, 13.8174}	{{1.15921, 8.09734}, {2.67299, 19.0531}}	{27.3618, 58.6807}	inf	32.	74.019	{3.85453, 175.958}	172.846	0.540285	newly	23.	39.36	{3., 82.}	115.043	0.499612	Reff	1.51231	1.34943	{0.804713, 2.02856}	30.0093	ar	663.	643.02	{220., 912.}	30.3409
5	R0	{1.53822, 8.05553}	{1.75329, 13.2993}	{{1.11461, 5.40945}, {2.75598, 19.0269}}	{29.6056, 77.811}	inf	44.	75.2769	{3.47661, 180.951}	130.578	0.5476	newly	31.	37.21	{3., 83.}	78.9355	0.484143	Reff	1.33671	1.16562	{0.748712, 1.67839}	30.2757	ar	621.	601.31	{197., 912.}	34.9968
6	R0	{1.69374, 17.0793}	{1.6471, 14.5899}	{{1.20096, 7.71529}, {2.13931, 20.}}	{20.6374, 23.8593}	inf	45.	87.3624	{23.8832, 171.007}	117.387	0.32193	newly	15.	39.88	{13., 73.}	171.6	0.374908	Reff	1.54638	1.08022	{0.745844, 1.3234}	32.2358	ar	662.	741.3	{466., 967.}	25.9909
7	R0	{1.42179, 11.8031}	{1.7757, 15.0156}	{{1.23088, 8.99124}, {2.52255, 19.7723}}	{30.9813, 37.2891}	inf	53.	99.6056	{31.4811, 204.249}	107.01	0.281329	newly	34.	43.76	{15., 82.}	59.1176	0.218729	Reff	1.16871	1.04083	{0.556896, 1.40539}	22.6488	ar	517.	799.36	{560., 1024.}	55.5513
8	R0	{1.46683, 6.95875}	{1.75766, 13.3961}	{{1.11228, 6.62607}, {2.55942, 18.743}}	{32.7695, 98.0205}	inf	33.	78.2816	{3.85453, 190.863}	179.836	0.530155	newly	39.	38.65	{4., 82.}	63.7179	0.438224	Reff	1.32308	1.23051	{0.781251, 1.78306}	29.7047	ar	653.	601.55	{214., 902.}	34.1424
9	R0	{1.34525, 11.505}	{1.70615, 14.4703}	{{1.20567, 7.99191}, {2.43686, 19.9561}}	{32.194, 40.0589}	inf	25.	83.3327	{29.8557, 177.109}	235.502	0.427809	newly	24.	39.76	{16., 77.}	82.75	0.23658	Reff	1.20669	1.05053	{0.557799, 1.41787}	24.0046	ar	532.	757.07	{480., 1013.}	46.1523
10	R0	{1.77958, 14.372}	{1.78897, 15.2117}	{{1.2477, 7.70414}, {2.38925, 20.}}	{21.0328, 27.3688}	inf	100.	115.309	{31.3067, 225.941}	64.1015	0.290204	newly	47.	50.92	{17., 96.}	51.3617	0.232277	Reff	1.3685	1.05007	{0.697612, 1.32912}	27.1662	ar	671.	811.5	{528., 1024.}	29.9136
11	R0	{1.60334, 19.9548}	{1.62479, 14.2314}	{{1.20584, 7.39048}, {2.24827, 20.}}	{20.464, 28.75}	inf	56.	72.3018	{22.2216, 132.927}	68.4133	0.2723	newly	27.	32.93	{12., 60.}	55.2963	0.220608	Reff	1.33398	0.862692	{0.51934, 1.14178}	35.5278	ar	617.	725.34	{469., 973.}	28.3339
12	R0	{1.68623, 7.58585}	{1.79799, 13.0227}	{{1.13754, 6.0993}, {2.73737, 18.4249}}	{26.7937, 80.531}	inf	50.	81.8386	{8.24928, 202.343}	118.	0.462169	newly	56.	44.15	{5., 97.}	57.6607	0.431776	Reff	1.4822	1.27258	{0.814482, 1.74375}	24.9394	ar	640.	623.71	{214., 899.}	33.1109
13	R0	{1.82689, 18.0022}	{1.7201, 14.2498}	{{1.23948, 7.35904}, {2.54462, 20.}}	{23.7645, 25.5048}	inf	85.	88.5919	{28.122, 171.434}	57.125	0.272461	newly	30.	41.66	{17., 69.}	61.6	0.206368	Reff	1.51084	1.0802	{0.648773, 1.42588}	31.7963	ar	673.	749.53	{503., 1002.}	25.8202
14	R0	{1.75911, 9.16883}	{1.76384, 13.3276}	{{1.07469, 7.10524}, {2.51743, 17.6755}}	{25.4992, 54.938}	inf	22.	80.6667	{4.99197, 191.076}	298.377	0.586636	newly	24.	43.	{3., 90.}	126.5	0.47132	Reff	1.63773	1.23964	{0.813514, 1.74375}	30.207	ar	661.	618.22	{213., 893.}	32.1815
15	R0	{1.46168, 11.3859}	{1.77043, 14.4711}	{{1.24222, 8.25849}, {2.61778, 19.984}}	{28.4288, 39.9576}	inf	59.	91.9788	{26.59, 178.708}	86.2329	0.272706	newly	40.	40.65	{17., 73.}	44.425	0.19745	Reff	1.10795	0.963359	{0.616863, 1.30685}	22.7822	ar	556.	790.53	{503., 1029.}	46.0558
16	R0	{1.53577, 11.7581}	{1.77644, 13.3176}	{{1.07469, 6.8071}, {2.51743, 17.6755}}	{30.8614, 32.0845}	inf	48.	82.5021	{4.99197, 195.555}	125.782	0.49209	newly	31.	43.77	{3., 95.}	94.7419	0.447476	Reff	1.38834	1.24508	{0.814482, 1.72673}	24.2925	ar	625.	621.8	{213., 893.}	35.104
17	R0	{1.70698, 9.72924}	{1.7697, 12.7918}	{{1.11164, 6.0993}, {2.73737, 18.8228}}	{26.8825, 49.6161}	inf	51.	79.2615	{8.24928, 202.343}	111.397	0.45558	newly	41.	43.46	{6., 97.}	70.1463	0.396582	Reff	1.46288	1.25971	{0.813514, 1.7415}	25.5738	ar	675.	609.07	{213., 899.}	31.6963
18	R0	{1.5973, 14.931}	{1.83086, 13.1929}	{{1.05743, 6.8071}, {2.73517, 17.5057}}	{31.7111, 22.0813}	inf	33.	90.0174	{7.16405, 212.929}	208.34	0.521471	newly	22.	47.63	{6., 97.}	153.591	0.479988	Reff	1.48229	1.22871	{0.807409, 1.70495}	28.0971	ar	632.	639.44	{214., 913.}	34.2848
19	R0	{1.87468, 17.004}	{1.68163, 14.9642}	{{1.20329, 7.72916}, {2.38134, 19.9824}}	{23.0372, 20.9621}	inf	109.	87.5375	{26.8096, 192.434}	50.2052	0.303871	newly	48.	39.05	{15., 73.}	44.9375	0.252872	Reff	1.37227	0.943141	{0.590631, 1.19928}	32.2309	ar	692.	750.82	{474., 998.}	24.185
20	R0	{1.84715, 12.531}	{1.65698, 14.0269}	{{1.17141, 6.64521}, {2.3731, 20.}}	{23.683, 33.6091}	inf	87.	84.7941	{24.8761, 193.158}	60.0461	0.30361	newly	54.	37.68	{13., 66.}	47.4815	0.295179	Reff	1.37797	0.909737	{0.626536, 1.19468}	34.4588	ar	661.	733.3	{511., 1005.}	23.8427
21	R0	{1.75938, 13.5959}	{1.83801, 13.0882}	{{1.07874, 6.31117}, {2.73737, 17.9172}}	{27.293, 26.3539}	inf	51.	89.7595	{8.98592, 211.507}	128.279	0.477879	newly	26.	47.16	{4., 97.}	124.	0.445525	Reff	1.52538	1.23792	{0.771241, 1.74375}	28.6963	ar	620.	649.1	{230., 907.}	34.0161
22	R0	{1.63456, 10.7469}	{1.68163, 14.7805}	{{1.20701, 7.32084}, {2.37926, 20.}}	{21.9058, 50.8332}	inf	101.	95.2758	{28.7609, 225.934}	59.8264	0.310206	newly	65.	41.79	{15., 84.}	51.3077	0.333587	Reff	1.27823	0.995652	{0.673467, 1.27871}	24.9114	ar	654.	752.53	{487., 1026.}	26.711
23	R0	{1.62702, 19.1392}	{1.63872, 13.277}	{{1.11228, 6.05643}, {2.30371, 19.255}}	{24.0277, 31.2281}	inf	73.	64.8495	{5.34355, 144.815}	67.6648	0.476813	newly	31.	31.4	{4., 63.}	67.2258	0.399631	Reff	1.28209	1.01121	{0.653594, 1.41937}	30.887	ar	600.	574.46	{225., 861.}	32.6733
24	R0	{1.70466, 12.1202}	{1.56861, 12.6474}	{{1.06836, 5.72868}, {2.1245, 18.834}}	{23.7929, 32.9478}	inf	59.	46.1048	{2.61721, 107.725}	60.689	0.504964	newly	37.	21.55	{2., 48.}	53.8649	0.499054	Reff	1.25293	0.835655	{0.466567, 1.11156}	37.1431	ar	613.	549.18	{225., 839.}	33.8564
25	R0	{1.61735, 11.3169}	{1.62341, 14.3506}	{{1.18613, 7.01274}, {2.27653, 20.}}	{21.7214, 45.3798}	inf	88.	64.5398	{10.0698, 162.933}	55.7078	0.397017	newly	70.	28.03	{7., 58.}	61.1571	0.535712	Reff	1.2583	0.833278	{0.490115, 1.10968}	34.7073	ar	681.	721.37	{474., 967.}	23.3906
26	R0	{1.56977, 12.3035}	{1.78283, 13.8731}	{{1.14096, 6.0993}, {2.67299, 19.1994}}	{28.2627, 33.1706}	inf	74.	75.9677	{3.91537, 175.555}	71.1036	0.487395	newly	43.	37.69	{4., 82.}	57.5581	0.46733	Reff	1.30605	1.2302	{0.759278, 1.77136}	29.4871	ar	594.	625.65	{244., 904.}	35.0657
27	R0	{1.46317, 5.8298}	{1.62947, 13.5049}	{{1.11365, 6.05643}, {2.24606, 19.255}}	{25.5736, 134.04}	inf	28.	65.0123	{4.92125, 145.431}	175.189	0.490641	newly	37.	31.67	{3., 65.}	58.7838	0.438996	Reff	1.28027	1.06682	{0.713138, 1.53048}	28.0874	ar	586.	562.63	{184., 831.}	32.8754
28	R0	{1.70002, 14.9806}	{1.77937, 13.2406}	{{1.07469, 6.55128}, {2.51743, 17.6755}}	{27.0376, 23.0361}	inf	49.	82.3617	{4.99197, 195.555}	122.786	0.490601	newly	27.	43.68	{3., 95.}	109.778	0.448857	Reff	1.54702	1.25376	{0.814482, 1.74375}	27.2395	ar	652.	617.24	{213., 896.}	33.7485
29	R0	{1.52729, 16.1014}	{1.69293, 14.7282}	{{1.20616, 7.70935}, {2.3168, 20.}}	{23.3716, 22.6958}	inf	86.	81.6806	{24.948, 158.254}	56.3309	0.293914	newly	43.	35.24	{13., 68.}	46.8837	0.266337	Reff	1.16379	0.905864	{0.530914, 1.19486}	25.9277	ar	592.	751.6	{494., 991.}	34.4338
30	R0	{1.72795, 13.5635}	{1.74171, 14.0946}	{{1.21458, 7.9141}, {2.38749, 19.9784}}	{21.945, 29.2935}	inf	101.	91.9532	{26.4339, 219.566}	56.6659	0.316231	newly	52.	41.75	{15., 85.}	46.8654	0.267894	Reff	1.1992	0.970907	{0.624222, 1.25463}	22.9726	ar	668.	783.78	{542., 1007.}	27.1018
31	R0	{1.51591, 18.1544}	{1.76208, 14.7353}	{{1.20143, 8.27076}, {2.57201, 20.}}	{27.2326, 23.7061}	inf	41.	87.9782	{25.1326, 165.795}	131.239	0.324245	newly	13.	40.72	{14., 67.}	215.538	0.432665	Reff	1.27185	1.06923	{0.589659, 1.37472}	24.9379	ar	541.	783.02	{507., 1017.}	49.0721
32	R0	{1.4282, 10.3534}	{1.73558, 13.7687}	{{1.207, 5.22934}, {2.39141, 20.}}	{30.0333, 51.2439}	inf	21.	88.797	{23.1714, 188.835}	324.515	0.503387	newly	8.	47.01	{15., 90.}	488.125	0.679669	Reff	1.31109	1.17803	{0.775504, 1.58578}	21.855	ar	521.	767.21	{481., 1005.}	51.5605
33	R0	{1.56693, 11.3972}	{1.7758, 13.1919}	{{1.07662, 6.0993}, {2.65407, 17.9172}}	{28.9091, 35.7076}	inf	51.	79.4707	{7.99636, 195.555}	114.873	0.487555	newly	36.	43.38	{4., 97.}	81.	0.437149	Reff	1.38203	1.27178	{0.813756, 1.74375}	24.6917	ar	695.	617.79	{214., 896.}	29.7741
34	R0	{1.50675, 19.8823}	{1.69845, 14.121}	{{1.22992, 7.00257}, {2.44921, 20.}}	{24.1959, 29.167}	inf	72.	65.2511	{16.648, 134.159}	56.1096	0.304305	newly	29.	29.44	{11., 56.}	47.5862	0.222597	Reff	1.0502	0.885105	{0.524749, 1.20365}	23.6409	ar	587.	751.06	{505., 1014.}	34.6848
35	R0	{1.72889, 7.76068}	{1.79234, 13.1901}	{{1.07662, 6.77341}, {2.51417, 17.8178}}	{25.801, 76.9815}	inf	36.	82.4344	{4.99197, 202.343}	174.786	0.515977	newly	45.	44.36	{3., 97.}	68.7111	0.467139	Reff	1.55946	1.24722	{0.798891, 1.83304}	28.6674	ar	650.	630.36	{213., 894.}	32.76
36	R0	{1.62644, 4.12303}	{1.9227, 12.8649}	{{1.19018, 6.25107}, {2.78218, 18.5502}}	{31.6373, 213.899}	inf	30.	94.4901	{7.33622, 199.278}	240.072	0.55178	newly	61.	54.72	{5., 101.}	53.8033	0.405695	Reff	1.31091	1.31164	{0.717083, 2.0885}	29.1592	ar	652.	683.32	{327., 912.}	30.3896
37	R0	{1.70878, 15.8072}	{1.60177, 14.2896}	{{1.20719, 7.13322}, {2.2703, 20.}}	{20.4602, 25.6629}	inf	96.	66.8601	{18.3117, 121.558}	50.3586	0.354371	newly	49.	29.21	{10., 54.}	49.7347	0.361716	Reff	1.21665	0.840146	{0.532601, 1.10595}	31.5877	ar	674.	731.67	{498., 961.}	21.2834
38	R0	{1.58967, 6.80568}	{1.82515, 13.4633}	{{1.11461, 6.78013}, {2.66419, 19.0531}}	{32.0488, 101.9}	inf	44.	90.6192	{6.60572, 205.666}	150.665	0.502159	newly	51.	44.13	{5., 97.}	57.1961	0.433671	Reff	1.3846	1.22456	{0.786219, 1.78306}	29.3832	ar	610.	629.76	{226., 908.}	37.7607
39	R0	{1.344, 8.19498}	{1.77324, 14.7647}	{{1.23961, 7.24881}, {2.61853, 20.}}	{35.7008, 88.7037}	inf	37.	102.224	{25.2964, 231.755}	190.975	0.405432	newly	32.	46.19	{16., 89.}	73.0313	0.254428	Reff	1.1545	1.03182	{0.688156, 1.34841}	22.3575	ar	551.	771.06	{488., 1027.}	45.9782
40	R0	{1.61509, 7.90825}	{1.81199, 13.0875}	{{1.14096, 6.83219}, {2.51743, 18.4249}}	{28.3175, 70.7902}	inf	38.	73.2164	{4.16493, 168.25}	138.849	0.482223	newly	48.	41.9	{3., 90.}	60.5	0.47557	Reff	1.39544	1.38549	{0.852404, 2.02903}	24.9409	ar	581.	639.72	{284., 893.}	36.3236
41	R0	{1.60017, 5.90605}	{1.74252, 13.4743}	{{1.11461, 6.05643}, {2.73517, 19.0269}}	{27.6944, 132.625}	inf	46.	77.1851	{3.47661, 180.951}	125.158	0.533392	newly	81.	38.31	{3., 83.}	57.0988	0.630653	Reff	1.38735	1.18837	{0.759278, 1.67839}	29.2837	ar	661.	596.5	{197., 905.}	32.1876
42	R0	{1.64436, 15.1337}	{1.77588, 13.875}	{{1.11228, 6.0993}, {2.59907, 19.2972}}	{26.9516, 25.9971}	inf	52.	81.9826	{3.91537, 185.478}	110.965	0.500386	newly	26.	38.94	{4., 83.}	94.4615	0.441673	Reff	1.38455	1.16487	{0.731069, 1.70824}	29.5807	ar	640.	632.51	{245., 902.}	30.4641
43	R0	{1.48917, 11.917}	{1.55674, 13.3647}	{{1.18176, 5.43945}, {2.08841, 20.}}	{18.2619, 36.013}	inf	67.	65.2896	{9.79204, 129.467}	57.7428	0.347753	newly	42.	29.91	{7., 58.}	48.0238	0.354857	Reff	1.16602	0.851802	{0.566528, 1.09066}	28.1818	ar	551.	696.49	{479., 948.}	33.2214
44	R0	{1.65577, 7.89317}	{1.811, 13.4736}	{{1.11228, 6.31117}, {2.59907, 19.0269}}	{27.3073, 79.0335}	inf	24.	76.8081	{3.85453, 182.522}	254.359	0.57524	newly	30.	38.89	{4., 81.}	80.5	0.450835	Reff	1.52331	1.26675	{0.793859, 1.94828}	32.2742	ar	576.	631.03	{219., 906.}	39.6406
45	R0	{1.6909, 12.3275}	{1.629, 12.9908}	{{1.13176, 5.71232}, {2.22212, 19.0443}}	{21.3643, 34.4109}	inf	89.	68.4673	{2.57648, 144.815}	61.7714	0.525863	newly	56.	33.27	{3., 65.}	50.7321	0.496487	Reff	1.29354	0.995023	{0.698749, 1.41937}	29.6164	ar	675.	580.31	{248., 841.}	27.6933
46	R0	{1.62351, 16.0698}	{1.73666, 14.9828}	{{1.24063, 8.96223}, {2.46366, 20.}}	{23.288, 24.2329}	inf	41.	89.6943	{28.6639, 171.74}	133.973	0.328699	newly	19.	40.38	{15., 66.}	121.368	0.306968	Reff	1.43681	1.06867	{0.545498, 1.50336}	30.8478	ar	591.	776.95	{527., 1027.}	35.8426
47	R0	{1.52372, 4.38599}	{1.80645, 13.1918}	{{1.09005, 6.74711}, {2.79269, 18.8445}}	{34.5777, 202.04}	inf	11.	80.2975	{6.295, 188.089}	643.939	0.753251	newly	26.	45.31	{4., 95.}	117.808	0.428386	Reff	1.44449	1.34712	{0.887318, 2.0885}	26.6472	ar	618.	620.07	{197., 914.}	36.1278
48	R0	{1.44306, 11.788}	{1.60368, 12.8051}	{{1.11228, 5.72868}, {2.22212, 19.0355}}	{24.8692, 36.5334}	inf	53.	58.6647	{2.81552, 134.885}	82.9521	0.5105	newly	34.	27.96	{2., 58.}	61.8824	0.464017	Reff	1.12126	0.966885	{0.613203, 1.44712}	28.2309	ar	534.	566.91	{223., 839.}	36.4213
49	R0	{1.63624, 11.3893}	{1.82941, 13.576}	{{1.11228, 6.8071}, {2.67299, 19.0269}}	{30.4277, 35.4092}	inf	71.	90.2832	{6.91464, 209.327}	88.8617	0.474639	newly	46.	42.74	{6., 96.}	59.087	0.41317	Reff	1.39244	1.19112	{0.760846, 1.77856}	31.623	ar	643.	634.17	{226., 911.}	34.4681
50	R0	{1.46113, 11.1322}	{1.70615, 13.4465}	{{1.22072, 5.56549}, {2.38419, 20.}}	{26.9997, 43.5127}	inf	34.	80.4363	{22.1491, 187.824}	151.185	0.329317	newly	19.	44.24	{14., 83.}	143.895	0.336327	Reff	1.33547	1.18658	{0.756294, 1.66462}	21.9289	ar	501.	761.13	{522., 1005.}	53.6906
